# 16S rRNA Gene Sequencing for Deciphering the Colorectal Cancer Gut Microbiome: Current Protocols and Workflows

**DOI:** 10.3389/fmicb.2018.00767

**Published:** 2018-04-27

**Authors:** Muhammad-Afiq Osman, Hui-min Neoh, Nurul-Syakima Ab Mutalib, Siok-Fong Chin, Rahman Jamal

**Affiliations:** UKM Medical Molecular Biology Institute, Universiti Kebangsaan Malaysia, Kuala Lumpur, Malaysia

**Keywords:** gut microbiome, metagenomics, next-generation sequencing, 16S rRNA gene, 16S rRNA gene sequencing, colorectal cancer, colorectal cancer screening

## Abstract

The human gut holds the densest microbiome ecosystem essential in maintaining a healthy host physiology, whereby disruption of this ecosystem has been linked to the development of colorectal cancer (CRC). The advent of next-generation sequencing technologies such as the 16S rRNA gene sequencing has enabled characterization of the CRC gut microbiome architecture in an affordable and culture-free approach. Nevertheless, the lack of standardization in handling and storage of biospecimens, nucleic acid extraction, 16S rRNA gene primer selection, length, and depth of sequencing and bioinformatics analyses have contributed to discrepancies found in various published studies of this field. Accurate characterization of the CRC microbiome found in different stages of CRC has the potential to be developed into a screening tool in the clinical setting. This mini review aims to concisely compile all available CRC microbiome studies performed till end of 2016 and to suggest standardized protocols that are crucial in developing a gut microbiome screening panel for CRC.

## Introduction

The human gut harbors an enormous, diverse, and dynamic microbiome, consisting primarily of bacteria and archaea, as well as fungi, protozoa and viruses. There are at least 100 trillion (10^14^) microbial cells in the human gut, almost outnumbering the eukaryotic cells that reside together (Whitman et al., 1998; Costello et al., 2012; Sender et al., 2016). The gut microbiome is known to play a vital role in health, contributing toward the host's energy harvest and storage via various metabolic functions (Gill et al., 2006). In good health, our gut microbiota is mainly subdivided into two categories; commensal symbionts and commensal pathobionts. Commensal bacteria has been acknowledged to be important for host physiology through provision of essential nutrients and providing protection against colonization by opportunistic pathogens (Hooper and Gordon, 2001).

The gut microbiota has been typically controlled by environmental factors such as adoption of westernized diet and lifestyle (David et al., 2014). Incidentally, there is growing evidence to suggest that environmental factors such as obesity and diet are associated with the pathogenesis of colorectal cancer (CRC). As the mechanism of sporadic CRC is still poorly understood, an individual's gut microbiome landscape may reflect his or her dietary patterns which can either promote or protect against CRC (Bultman, 2017).

Bacterial composition in the gut helps to maintain its host's mucosal and systemic immunity homeostasis, avoiding any immunity trigger that might lead to physiological impairment. A shift of gut commensal microbiota toward opportunistic pathogens is a condition designated as dysbiosis (Barman et al., 2008). A few studies suggested dysbiosis as the scenario that will impact numerous physiological functions and that this will serve as a primary driver for inflammation in the colon leading to increased risk for CRC (Nistal et al., 2015). Many recent studies have started to disclose that the gut microbiome plays a role in oncogenesis, where their interaction with the immune system might either maintain a healthy host or drive tumor progression (Gagliani et al., 2014).

Unlike gastric cancer which is solely associated with *Helicobacter pylori* infection, metagenomics studies showed that fecal and mucosal samples of CRC patients and non-CRC individuals are enriched in different microbiome composition (Uemura et al., 2001; Thomas et al., 2016; Flemer et al., 2017). Most studies reported *Bacteroides, Fusobacterium*, and *Peptostreptococcus* as the more prominent genera in CRC samples compared to controls (Table 1). CRC patients also showed an increase in the abundance of *Gemella, Parvimonas*, and *Porphorymonas* (Chen et al., 2012; Allali et al., 2015; Sinha et al., 2016).

**Table 1:** CRC-associated bacteria of different geographical locations.

**Publication**	**Location**	**CRC-associated Bacteria**
Baxter et al., 2016	Canada & USA	*F. nucleatum, P. assaccharolytica, P. stomatis, Gemella sp., Prevotella sp. P. micra*
Flemer et al., 2017	Cork, Ireland	*Bacteroides, Roseburia, Ruminococcus, Oscillibacter, Porphyromonas, Peptostreptococcus, Parvimonas, Fusobacterium*
Sinha et al., 2016/ Ahn et al., 2013	Maryland/New York, USA	*Fusobacterium, Porphyromonas, Atobium*
Allali et al., 2015	US & Spain	*US: Eikenella Spain: Fusobacterium, Bulleida, Gemella, Parvimonas, Campylobacter, and Streptococcus*
Burns et al., 2015	Minneapolis, USA	*Fusobacterium, Providencia*
Gao et al., 2015	Shanghai, China	*Bacteroides, Prevotella, Streptococcus, Lactococcus, Fusobacterium*
Nakatsu et al., 2015	Guanzhou & Hong Kong, China	*B. fragilis, Gemella, Parvimonas, Peptostreptococcus, Granulicatella*
Zeller et al., 2014	Heidelberg, Germany & Créteil, France	*F. nucleatum, P. asaccharolytica, B. fragilis, E. ventriosum, E. eligens C. symbiosum, S. salivarus*
Dejea et al., 2014	Marryland, USA	*Lactococcus, Leuconostoc, Comamonas*
Mira-Pascual et al., 2015	Spain	*Fusobacterium, Bacteroides, and Methanobacteriales*
Zackular et al., 2014	UK	*Fusobacterium, Porphyromonas, Lachnospiraceae, and Enterobacteriaceae*
Geng et al., 2013	China	*Roseburia*
Weir et al., 2013	Colorado, USA	*Acidaminobacter unclassified, Phascolarctobacterium unclassified, Citrobacter farmeri and Akkermansia muciniphila*
Wu et al., 2013	Beijing, China	*Bacteroides, Campylobacter and Fusobacterium*
Chen et al., 2012	China	*Peptostreptococcus, Parvimonas, Fusobacterium, Mogibacterium, Porphyromonas*
Kostic et al., 2012	Spain	*Fusobacterium*
Wang et al., 2012	Shanghai, China	*Enterococcus, Escherichia/Shigella, Klebsiella, Streptococcus* and *Peptostreptococcus*
Marchesi et al., 2011	Netherland	*Slackia, Collinsella*
Sobhani et al., 2011	Paris, France	*Bacteroides/Prevotella*

In conjunction with the acknowledgement of gut microbiome contribution toward health and disease, developments in next-generation sequencing provided many breakthroughs for taxonomic, phylogenetic or functional profiling of the gut microbiome. A metagenomics approach toward gut microbiome profiling will confer the added advantage of not only metagenome community characterization, but also provides answers on its physiological impact to the human host. Furthermore, via metagenomics sequencing, measurements of bacterial taxa abundance within a sample as well as identification of dysbiosis events within its tumor microenvironment could be carried out. Indeed, the Metagenomics of Human Intestinal Tract (MetaHIT) project was initiated to study associations between genes of the gut microbiome with health and disease (Qin et al., 2010).

**Figure 1:**
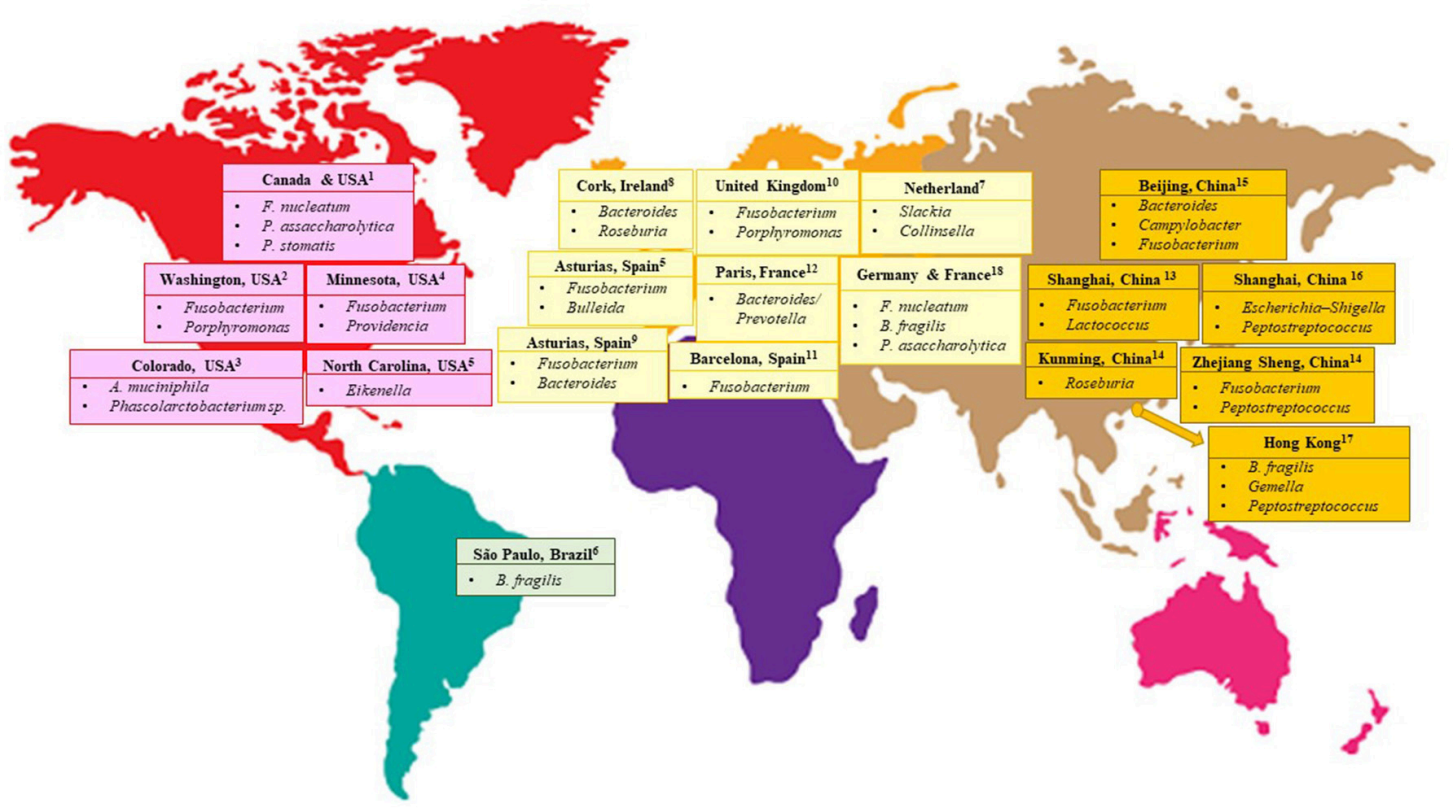
CRC-associated bacteria from different geographical locations of the world. ^1^(Baxter et al., 2016) ^2^(Ahn et al., 2013; Sinha et al., 2016) ^3^ (Weir et al., 2013) ^4^(Burns et al., 2015) ^5^(Allali et al., 2015) ^6^(Thomas et al., 2016) ^7^(Marchesi et al., 2011) ^8^(Flemer et al., 2017) ^9^(Mira-Pascual et al., 2015) ^10^(Zackular et al., 2014) ^11^(Kostic et al., 2012) ^12^(Sobhani et al., 2011) ^13^(Gao et al., 2015) ^14^(Geng et al., 2013) ^15^(Wu et al., 2013) ^16^(Wang et al., 2012) ^17^ (Nakatsu et al., 2015) ^18^(Zeller et al., 2014).

Nevertheless, despite similar approaches being used in several gut microbiome studies on CRC patients, dissimilarities in results were still apparent (Table 1). These dissimilarities may either be caused by differences in the patients' dietary customs specific to a certain geographical location (Figure 1) or caused by the technical aspects of next-generation sequencing experiments due to variations in sample handling and processing, and bioinformatics analysis pipelines. Lack of standardization in human microbiome studies may cause repetitive discrepancies if no baseline protocol is available. To this end, recently, The Microbiome Quality Control (MBQC) project (http://www.mbqc.org/)has been initiated to expand and encourage open sharing of standard operating procedures and best practices in the metagenomics field (Sinha et al., 2015).

To elucidate the gut microbiome landscape in tumor microenvironments, specific study design and experiment protocols are required for metagenomics sequencing and analyses. In this review, we discuss the culture-independent application of 16S rRNA gene sequencing in cataloguing the CRC gut microbiome. This review will also cover the assessment of 16S rRNA gene sequencing of the CRC gut microbiome, covering steps from nucleic acid extraction, sample preparation, selection of hypervariable regions, sequencing platforms, and the determination of algorithms for bioinformatics analyses, which we believe will provide an insight into CRC gut microbiome characterization via the next-generation sequencing approach.

## High-throughput microbiome sequencing

Advances in high-throughput sequencing technologies have enabled researchers to explore microbiome complexity associated with the human body and diseases. In the past decade, application of high-throughput DNA sequencing to profile the genomic composition of a microbial community in a culture-independent manner has expanded immensely. This high resolution molecular sequencing technique, designated as metagenomics, can be further sub-divided into two different approaches, namely 16S rRNA gene and shotgun metagenomics sequencing. The 16S rRNA gene sequencing approach relies on sequencing of the 16S ribosomal RNA (rRNA) gene as the genetic marker to study bacterial phylogeny and taxonomy. This genetic marker contains conserved hypervariable regions which can be used for bacteria identification. Selection of hypervariable regions for sequencing as well as amplicon primer design are important for 16S rRNA gene sequencing as these factors might contribute to differences in the results.

On the other hand, whole genome or shotgun metagenomics sequencing, an alternative to 16S rRNA gene sequencing, refers to massive parallel sequencing of DNA samples. This technique also identifies gene functions of the sequenced microbiome. Shotgun metagenomics sequencing involves random fragmentation of DNA, sequencing of these fragments, followed by reconstruction and assembly of overlapping sequences into a continuous sequence (Fraher et al., 2012). Researchers have used shotgun metagenomics sequencing to discover interactions between microbiota and its host. The disadvantages of this technique are that it is costly and it produces a huge amount of data that requires advanced bioinformatics analyses.

## 16S rRNA gene sequencing and its impact on human gut microbiome

Many studies have employed 16S rRNA gene sequencing to profile the gut microbiota composition (Gill et al., 2006; Huse et al., 2012; Yatsunenko et al., 2012). The 16S small subunit ribosomal gene is an exclusive housekeeping gene in prokaryotes which can be used to determine microbial communities within samples; it is highly-conserved and contains hypervariable regions ranging from region V1 to V9. Sequencing of the 16S rRNA gene requires amplification of a selected variable region via PCR using a variety of “universal” primers followed by sequencing. The V4 region of 16S rRNA gene has been highly recommended as the gold standard for profiling of human gut microbiome by the MetaHIT consortium (Qin et al., 2010; Lozupone et al., 2013). Besides selection of a suitable hypervariable region, compatibility of amplification fragment lengths with the read length capacity of the intended sequencing platform has to be confirmed.

The number of microbiome studies has increased enormously in concert with technological developments of DNA sequencing that facilitate culture- and cloning-free analyses. When it was first released in 2005, the first next-generation sequencer (Roche 454 pyrosequencer), can only sequence ~120 bases of bacterial genome in a single run (Margulies et al., 2005). In recent years, this technology has enabled the coverage of up to 1000 bp and can span multiple hypervariable regions of the 16S rRNA gene. Due to its high resolution and cost-effective approach, 16S rRNA gene sequencing has become the commonest approach for microbial community profiling of the human gut.

## 16S rRNA gene sequencing as a molecular screening tool for CRC

Guaiac fecal occult blood test (gFOBT) and the immunochemical-based fecal occult blood test (iFOBT/FIT) are current screening tools for CRC via detection of gastrointestinal bleeding. Nevertheless, these tests are not specific for CRC as pathologies such as ulcerative colitis and polyps could also cause bleeding in the gut. Gut microbiome profiling studies based on 16S rRNA gene sequencing has identified bacterial genus frequently associated with CRC, including *Fusobacterium, Bacteroides*, and *Peptostreptococcus* (Nakatsu et al., 2015; Baxter et al., 2016; Flemer et al., 2017; Table 1). In particular, the role of *F. nucleatum* in CRC tumorigenesis and metastasis has been consistently reported in animal models and cell culture experiments (Kostic et al., 2013; Rubinstein et al., 2013). Recent, additional studies confirmed the importance of *F. nucleatum* in CRC, where the bacteria was shown to modulate tumorigenesis in the colon via miRNA-21 expression, which subsequently suppresses the immune response and activate oncogenic pathways (Nosho et al., 2016; Yang et al., 2017). Zackular et al's study demonstrated the feasibility of using microbial biomarkers such as *F. nucleatum* for CRC screening (Zackular et al., 2014). In addition, Liang et al established a species-level microbiome panel that could distinguish between CRC patients and healthy individuals of the Hong Kong population with greater accuracy and sensitivity than the current screening kit (Liang et al., 2017). Recently, it has been suggested that FOBT coupled with 16S rRNA gene sequencing will serve as a better screening approach for CRC, where stool-containing buffer samples from the FOBT kits could be used for sequencing (Liang et al., 2017; Taylor et al., 2017). To this end, standardized workflows for 16S rRNA gene sequencing will be crucial to produce results which are accurate and reproducible.

## Sample collection and storage

An important but seldom emphasized aspect of 16S rRNA gene sequencing studies is sample integrity. For gut microbiome profiling studies, biopsy, surgical tissues and stool samples are the common biospecimens collected for characterization. Among these, stool samples were the first to be used for 16S rRNA gene sequencing to study the CRC microbiome. Subsequent studies used swabs, surgical or biopsy tissues as the starting material with different sample handling procedures. Minimization of sample contamination ensures that each biospecimen retains as much as possible of its original microbiome and contributes toward accurate results in gut microbiome profiling. Nevertheless, studies comparing methods of biospecimen handling remain few. Many published studies did not comprehensively disclose methods used for sample handling. Sobhani et al published the first 16S rRNA gene sequencing study on CRC microbiome in 2011. Their protocol used stool samples; samples were placed in a sterile carrier box, transported to the laboratory within 4 h and later stored at −20°C prior to DNA extraction. Subsequent studies published improvements in handling the biosamples and transportation protocols, including steps such as transportation of samples on ice (Wu et al., 2010) and storage at −80°C to preserve sample integrity (Consortium, 2012).

Stool samples require non-invasive techniques for collection and they are sufficient for researchers to obtain an overview of the gut microbiome spectrum in our colon. These samples could be studied using leftover stool-containing buffer from used FOBT and FIT cartridges (Baxter et al., 2016; Taylor et al., 2017). However, stool samples might not be able to provide a clear picture for studies exploring site-specific microbiome which would require a tissue biopsy. On the other hand, despite the invasiveness of tissue biopsy, these samples would enable in-depth studies into tumor-specific microbiota in comparison to stool sampling.

Short and long-term storage conditions post-transportation of samples is important to minimize differences in the microbiome spectrum caused by storage conditions. Sample transportation and storage conditions for fecal samples used in CRC gut microbiome characterization have been studied. Low temperature transportation, such as on ice or dry ice, ethanol-stored and long-term storage in −80°C are strongly recommended to maximize microbiome recovery within the sample prior nucleic acid extraction (Choo et al., 2015; Fouhy et al., 2015; Gorzelak et al., 2015; Blekhman et al., 2016). This also applies to biopsy and surgical tissue samples. Tissue handling protocols such snap-freezing cryovials in liquid nitrogen or usage of RNAlater medium will ensure optimum conditions for sample storage prior to nucleic acid extraction, leading to better microbiome recovery.

From the available literature, seven studies on CRC profiled the fecal microbiome, while others used colon tissue samples for their analyses (Table 1). Some studies investigated both fecal and tissue samples from the same individual to differentiate the microbiome compositions (Table 1). The authors from these studies applied various approaches for sample handling and storage of the biospecimens. For studies using fecal samples, samples were all transported fresh and on ice to the research laboratory in <24 h; all samples except the ones used in a particular study were stored in −80°C. For the exception, DNA was directly extracted from samples and stored at −20°C until library preparation (Weir et al., 2013). On the other hand, for studies using tissue samples as the starting material, samples were usually snap-frozen in liquid nitrogen. Flemer et al. and Dejea et al. chose to use RNAlater as a preservative medium to ensure maximum recovery of nucleic acids; while the studies by Burns et al. and Gao et al. performed nucleic acid extraction right after sample collection (Table 2). Overall, perhaps due to the fact that 16S rRNA gene sequencing studies are still new, long-term effects of sampling and storage conditions for gut microbiome specimens have not been described.

**Table 2:** Published CRC 16S rRNA sequencing for gut microbiome studies, 2011–2016.

**Publication**	**Sample Type**	**No. of CRC patients**	**Tissue samples**	**Sample handling**	**Sample storage**	**DNA Extraction kit**	**Hypervariable region**	**Sequencing Platform**	**OTU Classification Database**	**No. of Reads for Downstream/Total Reads**	**Bioinformatics Pipeline**
Thomas et al., 2016	Biopsy Tissue	18	Non-cancer (18)	N/A	N/A	Phenol-Chloroform & Ethanol Precipitation	V4/V5	Ion Torrent PGM	RDP	17, 414 reads/sample	QIIME
Baxter et al., 2016	Stool	120	Adenoma (198) Normal (172)	Prior bowel prep, ship on ice next day	−80°C	MoBio Kit, 50 mg	V4	Illumina MiSeq	RDP	10, 000 reads/sample	MOTHUR
Flemer et al., 2017	Stool, Surgical Tissue, Biopsy Tissue	59	Adenoma (21), Control (56)	RNAlater (4°C, 12H)—Tissue, Ice box—Fecal (prior bowel prep)	−20°C (Tissue), −80°C (Fecal)	QIAGEN AllPrep DNA/RNA kit plus with glass bead homogenization)	V3/V4	Illumina MiSeq	RDP	5, 000 reads/sample	QIIME
Sinha et al., 2016/ Ahn et al., 2013	Stool	42	Normal (89)	Dry ice	−40°C	MoBio PowerSoil DNA Isolation kit	V3/V4	454 Pyro sequencing	Greengenes	5, 000 reads/sample	QIIME
Allali et al., 2015	Surgical Tissue	90	Adjacent-Normal	Snap-frozen in Isopentane	−80°C	QIAGEN Dneasy Blood and Tissue with Lysozyme in ATL buffer	V1/V2	454 Pyro sequencing	Greengenes	719 reads/sample	QIIME
Burns et al., 2015	Surgical Tissue	44	Adjacent-Normal	Flash-frozen Liquid Nitrogen	Not stored	QIAzol, Sonicate Water bath @65°C	V5/V6	Illumina MiSeq	Greengenes	5, 000 reads/sample	QIIME
Gao et al. 2015	Surgical Tissue	30	Adjacent-Normal, Normal (31)	N/A	Not stored	MoBio PowerSoil DNA Isolation kit	V3	454 Pyro sequencing	RDP, Greengenes, ITS Fungus, FGR	17, 018 reads/sample	QIIME
Nakatsu et al., 2015	Biopsy Tissue	52	Adenoma (47), Normal (61)	Flash-frozen Liquid Nitrogen	−80°C	QIAGEN QIAamp Mini kit with beads beating & lysozyme	V1-V4	454 Pyro sequencing	Greengenes	1, 000 reads/sample	MOTHUR
Zeller et al., 2014	Stool & Surgical tissue	89	Adjacent-normal (48), Normal (75)	Flash-frozen liquid nitrogen	−80°C	GNOME® DNA Isolation Kit	V4	Illumina MiSeq	SILVA	10, 000 reads/sample	MOTHUR
Dejea et al., 2014	Surgical & Biopsy tissue	23	Adenoma (2), Normal (22)	RNAlater	−80°C	QIAGEN QIAamp Stool DNA kit	V3-V5	454 Pyro sequencing	RDP	N/A	QIIME
Mira-Pascual et al., 2015	Stool & Biopsy tissue	7	Adenoma (11), Normal (10)	N/A	−80°C	Machery-Nagel DNA kit with glass beads	V1/V2	454 Pyro sequencing	RDP	Biopsy/stool−6, 000 reads/sample, Biopsy-3groups−18, 000 reads/sample	MOTHUR
Zackular et al., 2014	Stool	30	Adenoma (30), Normal (30)	On ice (next day)	−80°C	MoBio PowerSoil DNA Isolation kit	V4	Illumina MiSeq	RDP	25, 993 reads/sample	MOTHUR
Geng et al., 2013	Biopsy Tissue	8	Adjacent-normal	Flash-frozen Liquid Nitrogen	−80°C	QIAGEN QIAamp DNA Mini kit with Silica beads	V1/V2	454 Pyro sequencing	BLAST	1, 400 reads/sample	QIIME
Weir et al., 2013	Stool	10	Normal (11)	Transport <24 h	N/A	MoBio PowerSoil DNA Isolation kit	V4	454 Pyro sequencing	Greengenes	125 sequence/sample	MOTHUR
Wu et al., 2013	Stool	20	Normal (20)	Frozen	−80°C	QIAGEN QIAamp Stool DNA kit	V3	454 Pyro sequencing	RDP	N/A	MOTHUR
Chen et al., 2012	Surgical Tissue, Stool & Swab	47	Normal (9)	N/A	−80°C	QIAamp DNA Mini/Stool kit with lysozyme	V1-V3	454 Pyro sequencing	N/A	N/A	MOTHUR
Kostic et al., 2012	Surgical tissue	95	Adjacent-Normal	Flash-frozen Liquid Nitrogen	−80°C	N/A	V3-V5	454 Pyro sequencing	RDP	N/A	MOTHUR
Wang et al., 2012	Stool	46	Normal (56)	Frozen	−80°C	Bead-beating + Phenol-Chloroform Purification	V3	454 Pyro sequencing	RDP	N/A	DOTUR
Marchesi et al., 2011	Surgical tissue	6	Adjacent-Normal	N/A	−80°C	QIAGEN AllPrep DNA/RNA kit	V1-V3	454 Pyro sequencing	RDP	N/A	MOTHUR
Sobhani et al., 2011	Stool	6	Normal (6)	N/A	N/A	GNOME® DNA Isolation kit with Silica beads	V3/V4	454 Pyro sequencing	BLAST	N/A	R Package: VEGAN

*N/A, Data/method was not described/stated*.

## Methods in nucleic acid extraction for perceiving the gut microbiome composition

Nucleic acid extraction of samples is a simple but critical step in microbiome studies. In recent years, a few debates have risen about the best isolation protocol which will give the most accurate representation of the microbial spectrum. Most isolation protocols comprise of three basic steps that include cellular lysis, non-DNA macromolecule elimination together with DNA detachment and collection. In general, cell lysis protocols have received the most scrutiny, as complete cell disruption achieved from either enzymatic and/or mechanical processes will enable subsequent comprehensive DNA isolation, and vice versa. Gram positive organisms require stronger lysis conditions due to their thicker cell walls, unlike gram negative organisms which require only gentle lysis (Brown et al., 2015). Several studies have been carried out with modifications of the nucleic acid extraction protocols compared with earlier published studies. These modifications include incorporation of additional procedures such as mechanical homogenization of cells with glass or silica beads, enzymatic lysis reaction with lysozyme, or a combination of both mechanical and enzymatic reactions (Table 2). Once cellular lysis has been accomplished, DNA clean-up, concentration, and elution were routinely carried out.

On the other hand, there were studies that achieved success in microbiome profiling without modifications toward standard lysis protocols (Table 2). Nevertheless, it should be noted that no isolation protocol works equally well on different sample types or produces completely unbiased results. The prevention of sample contamination during nucleic acid extraction is also vital to eliminate DNA from non-indigenous microbes. Proper sample handling such as working in clean laboratory environments and using commercially available DNA/RNA-free nucleic acid extraction reagents will decrease the risk of contamination. The operator should also don proper attire, gloves and face mask to protect samples from contamination with their own microbiota.

## Selection of universal 16S rRNA gene primers, sequencing technologies and databases

As described in an earlier section of this manuscript, the 16S rRNA gene consists of nine hypervariable conserved regions (V1 to V9) separated by ten highly conserved regions (Cox et al., 2013). The first 16S rRNA gene sequencing of CRC gut microbiome was completed 6 years ago using stool samples from 6 CRC patients (Sobhani et al., 2011). The study targeted the 16S rRNA V3/V4 regions, while its sequencing analyses relied on the database from the Ribosomal Database Project (RDP) using the RDP classifier (Cole et al., 2005). Similar studies targeting the same hypervariable regions were carried out a few years later using larger sample sizes and different biospecimens such as endoscopic biopsies and surgical tissues (Ahn et al., 2013; Flemer et al., 2017). Only very few studies were carried out to compare sequencing results obtained using primers targeting different hypervariable regions; most studies were done via sequencing of either one of the V3 or V4 regions, otherwise, a combination of two or more 16S rRNA gene hypervariable regions, whereby the most commonly used were the V3/V4 regions. Nevertheless, one study showed that while choice of primers had considerable effect, usage of matched primers on different sequencing platforms yielded little difference in results. (Tremblay et al., 2015). Differences obtained from the results of the various studies are conceivably due to factors such as selection of 16S rRNA gene primers and number of sequencing reads produced, as well as differences in classification techniques and bioinformatics analysis parameters. There is currently still no consensus on the best approach (Caporaso et al., 2011; Mizrahi-Man et al., 2013; Zheng et al., 2015).

Earlier gut microbiome studies in CRC were performed using the Roche 454 pyrosequencing technology (Table 2). Recently, the use of the Illumina MiSeq sequencer with paired-end reads and enhanced sequencing chemistry for microbiome studies has also increased (Caporaso et al., 2012). A study has been carried out to compare sequencing results produced between benchtop sequencers commonly used for 16S rRNA gene sequencing studies, including the Illumina MiSeq, Ion torrent Personal Genome Machine (PGM) and 454 GS Junior. Illumina MiSeq was found to generate data of the highest quality with almost no indel error compared to the other platforms (Loman et al., 2012).

Another important parameter in microbiome research is bioinformatics analysis. Most CRC microbiome studies rely on either the Greengenes or RDP database (Table 1; DeSantis et al., 2006; Cole et al., 2009). To the best of our knowledge, only RDP and SILVA are frequently updated; the Greengenes database has not been updated since 2013. On the other hand, the RefSeq Targeted Loci has been proposed to be the “gold standard” by the National Centre for Biotechnology Information (NCBI) for 16S rRNA gene sequencing data analysis (Tatusova et al., 2015). The NCBI database is also recommended for both 16S rRNA gene and shotgun metagenomics sequencing studies (Balvočiute and Huson, 2017). In addition, the SILVA database which covers phylogenies for small subunit rRNAs (16S for prokaryotes and 18S for eukaryotes) is also a resource for—aligning and/or quality-checking of rRNA sequence data (Pruesse et al., 2007; Quast et al., 2013). Recently, the EzBioCloud Genome database (previously known as EzTaxon) has been officially released. This database is well-curated; however, as it was recently launched, the database has yet been adapted in any 16S rRNA gene sequencing studies (Yoon et al., 2017). Despite the availability of numerous databases for 16S rRNA gene sequencing data analysis, a single, standardized database for this purpose is still unavailable.

The selection of appropriate computational tools for 16S rRNA gene sequencing dataset analysis is also crucial. Three commonly used bioinformatics pipelines were evaluated to determine the most precise tool available for unraveling the microbiome landscape via 16S rRNA gene sequencing (Plummer et al., 2015). Nevertheless, the comparison of findings from currently available studies showed that different results were obtained when analyses were run using different software or different databases. For all CRC gut microbiome studies carried out so far, most datasets were analyzed using Quantitative Insight into Microbial Ecology (QIIME) and MOTHUR (Schloss et al., 2009; Caporaso et al., 2010). Both tools were found to be precise for 16S rRNA gene sequencing dataset analysis.

## Concluding remarks and future perspectives

Initiatives on method standardization to study the human microbiome has been proposed by many research organizations such as the International Human Microbiome Standard (IHMS), MBQC and the well-known HMP project which acts as a baseline reference. In this mini review, we compiled and concisely compared all CRC 16S rRNA gene sequencing studies that have been carried out until 2016. As far as we know, no study is completely similar to another in terms of sample type and laboratory transfer, sequencing platform and primers, and bioinformatics database and analysis; therefore, the reproducibility of results obtained from a specific workflow could not be determined. Nevertheless, from our compilation, we found that most studies used the following workflow for CRC 16S rRNA gene sequencing: DNA extraction with mechanical homogenization, sequencing of the 16S rRNA V3/V4 regions, OTU picking using either the QIIME or MOTHUR software, and microbial classification against the Greengenes or RDP database. This commonly-used workflow was found to provide good quality sequencing reads and a comprehensive profile of CRC-associated gut microbiome. The reproducibility of 16S rRNA gene sequencing results from a specific workflow could only be tested via replicate experiments using identical workflow on the same DNA sample; this research approach could be further explored in future studies to build a sensitive, specific and non-invasive CRC molecular screening tool based on 16S rRNA gene sequencing.

## Author contributions

M-AO and HN drafted and wrote this manuscript. HN, N-SA, S-FC, and RJ were responsible for idea conception, critical evaluation, and manuscript review.

### Conflict of interest statement

The authors declare that the research was conducted in the absence of any commercial or financial relationships that could be construed as a potential conflict of interest.
